# Possible effect of the early administration of tranexamic acid on myocardial injury in patients with severe trauma

**DOI:** 10.1007/s11239-023-02898-4

**Published:** 2023-10-15

**Authors:** Alexandra Stroda, Simon Thelen, René M’Pembele, Theresa Tenge, Carina Jaekel, Erik Schiffner, Dan Bieler, Michael Bernhard, Ragnar Huhn, Giovanna Lurati Buse, Sebastian Roth

**Affiliations:** 1 Department of Anesthesiology, Medical Faculty, University Hospital Duesseldorf, Duesseldorf, Germany; 2 Department of Orthopedics and Trauma Surgery, Medical Faculty, University Hospital Duesseldorf, Moorenstr. 5, 40225 Duesseldorf, Germany; 3 Department of Trauma Surgery and Orthopedics, Bundeswehrzentralkrankenhaus, Koblenz, Germany; 4 Emergency Department, Medical Faculty, University Hospital Duesseldorf, Duesseldorf, Germany; 5 Department of Anesthesiology, Kerckhoff-Klinik, Bad Nauheim, Germany

**Keywords:** Tranexamic acid, Myocardial injury, Multiple trauma, Troponin

## Abstract

Hemodynamic stabilization plays a crucial role in the treatment of patients suffering from severe trauma. Current guidelines recommend the early administration of tranexamic acid (TXA) for bleeding control. While less blood loss can result in less end-organ damage, including myocardial injury, TXA also exhibits prothrombotic effects with potentially adverse myocardial effects. The aim of this study was to investigate the association between the administration of TXA and myocardial injury in patients with severe trauma. We conducted a monocentric cohort study including severely injured patients ≥ 18 years [defined by Injury severity score (ISS) ≥ 16], who were admitted to a tertiary care hospital between 2016 and 2019. Primary outcome measure was myocardial injury according to the fourth Universal Definition (= high sensitive troponin T ≥ 14 ng/l). Secondary endpoints were in-hospital major adverse cardiovascular events (MACE) and mortality. Main exposure was defined as administration of TXA during prehospital period. We conducted multivariate logistic regression models including predefined covariables. A total of 368 patients were screened. Among the 297 included patients (72% male, age. 55?21 years), 119 (40%) presented myocardial injury at hospital arrival. TXA was administered to 20/297 (7%) patients in the prehospital setting, and in 96/297 (32%) patients during pre-or in-hospital period. MACE incidence was 9% (26/297) and in-hospital mortality was 26% (76/297). The adjusted odds ratios (OR) for prehospital TXA and myocardial injury, MACE and mortality were 0.75 [95% confidence interval (CI): 0.25–2.23], 0.51 [95%CI: 0.06–4.30] and 0.84 [0.21–3.33], respectively. In the present cohort of patients suffering from severe trauma, prehospital TXA did not affect the incidence of myocardial injury.

**Supplementary Information:**

The online version contains supplementary material available at 10.1007/s11239-023-02898-4.

## Introduction

Hemodynamic stabilization plays a crucial role in the treatment of patients suffering from severe trauma. According to current guidelines, the early administration of tranexamic acid (TXA) for bleeding control is recommended in addition to permissive hypotension [1, 2]. TXA is a synthetic derivative of the amino acid lysine that acts by inhibiting the interaction between plasminogen and fibrin, reducing hyperfibrinolysis and consecuently reducing blood loss [3]. Large randomized trials have shown beneficial effects of TXA for early bleeding control [2, 4]. Less blood loss may lead to less end organ damages, i.e. myocardial injury, due to an optimization of oxygen supply and demand. On the other hand, TXA may have prothrombotic effects, especially when hyperfibrinolysis is not present. In those patients, TXA could possibly be associated with increased myocardial injury for example by microcirculatory thrombosis or microcirculatory disorders in the coronary artery system. A previous large randomized controlled trial could not show an association between TXA administration in patients with severe trauma and vasoocclusive myocardial infarction [2]. In a large-scale clinical study in noncardiac surgery patients, the boundary for non-inferiority of TXA for cardiovascular complications including myocardial was not reached [5]. The association between the administration of TXA and myocardial injury in patients with severe trauma has not been investigated. The aim of this study was to address the questions: (1) In patients with severe trauma, is prehospital TXA administration independently associated with myocardial injury? (2) in patients with severe trauma, is prehospital TXA administration independently associated with MACE or mortality?

## Methods

We conducted a monocentric cohort study with a retrospective design, approved by the local ethics committee of the Heinrich Heine University Duesseldorf (Reference nr. 2020 − 1122). All handling of personal data complied with the General Data Protection Regulation (EU) 2016/679.

### Study population

Consecutive severely injured adult patients ≥18 years (severe trauma = Injury Severity Score (ISS) ≥ 16), admitted to the resuscitation room of the University Hospital Duesseldorf between 01/2016 and 12/2019 were included. Patients without troponin measurement upon arrival and with insufficient documentation regarding the administration of TXA were excluded.

### Outcome measures and explanatory variable

Primary endpoint was the incidence of myocardial injury at presentation, defined by the fourth Universal Definition of myocardial infarction (= high sensitive Troponin T (hsTnT) ≥ 14 ng/l (Roche Diagnostics, Elecsys®) [6]. Secondary endpoints were (1) the incidence of major adverse cardiovascular events (MACE) during the initial hospital stay, including nonfatal cardiac arrest, acute myocardial infarction, new onset of cardiac arrhythmias and stroke, and (2) in-hospital mortality. MACE as composite endpoint was assumed if one of the diagnoses listed above was mentioned in the patient’s medical record. Independent variable in this study was the prehospital administration of TXA. The following covariables were chosen for risk adjustment of the logistic regression model: patients related covariates: age per year, sex, history of coronary artery disease (CAD), diabetes mellitus; trauma related covariates: hypotension in the resuscitation room (mean arterial pressure, MAP < 65mmHg), ISS (continuous),, thorax trauma, hemoglobin (continuous) measured in the resuscitation room. The choice of covariables was based on literature search and covariables were included when there was evidence for a potential association with myocardial injury and/or MACE particulary in the noncardiac surgery setting, as there is still lacking evidence for the trauma setting [7–10].

### Statistical analysis

For statistical analysis we used SPSS version 27.0. Complete case analysis was done. Continuous data are presented as median and corresponding interquartile range (IQR) or mean ± standard deviation (SD), whereas categorical data are shown as absolute numbers (percent). The independent association between administration of TXA and myocardial injury, as well as MACE and mortality, was determined using multivariate logistic regression (including forced entry of predefined covariables). To avoid overfitting, in presence of 119 events, we included 8 covariates for the main analysis [11].

### Sensitivity analysis

We conducted the following sensitivity analyses with slightly altered independent variables or endpoints to further analyse the association between TXA administration and myocardial injury: (1) Early administration of TXA, defined as administration either in the prehospital setting or in the resuscitation room, (2) myocardial injury on day one after admission, defined as hsTnT ≥ 14ng/l, (3) myocardial injury on day two after admission, defined as hsTnT ≥ 14ng/l. Covariables remained as mentioned above.

## Results

**Fig. 1:**
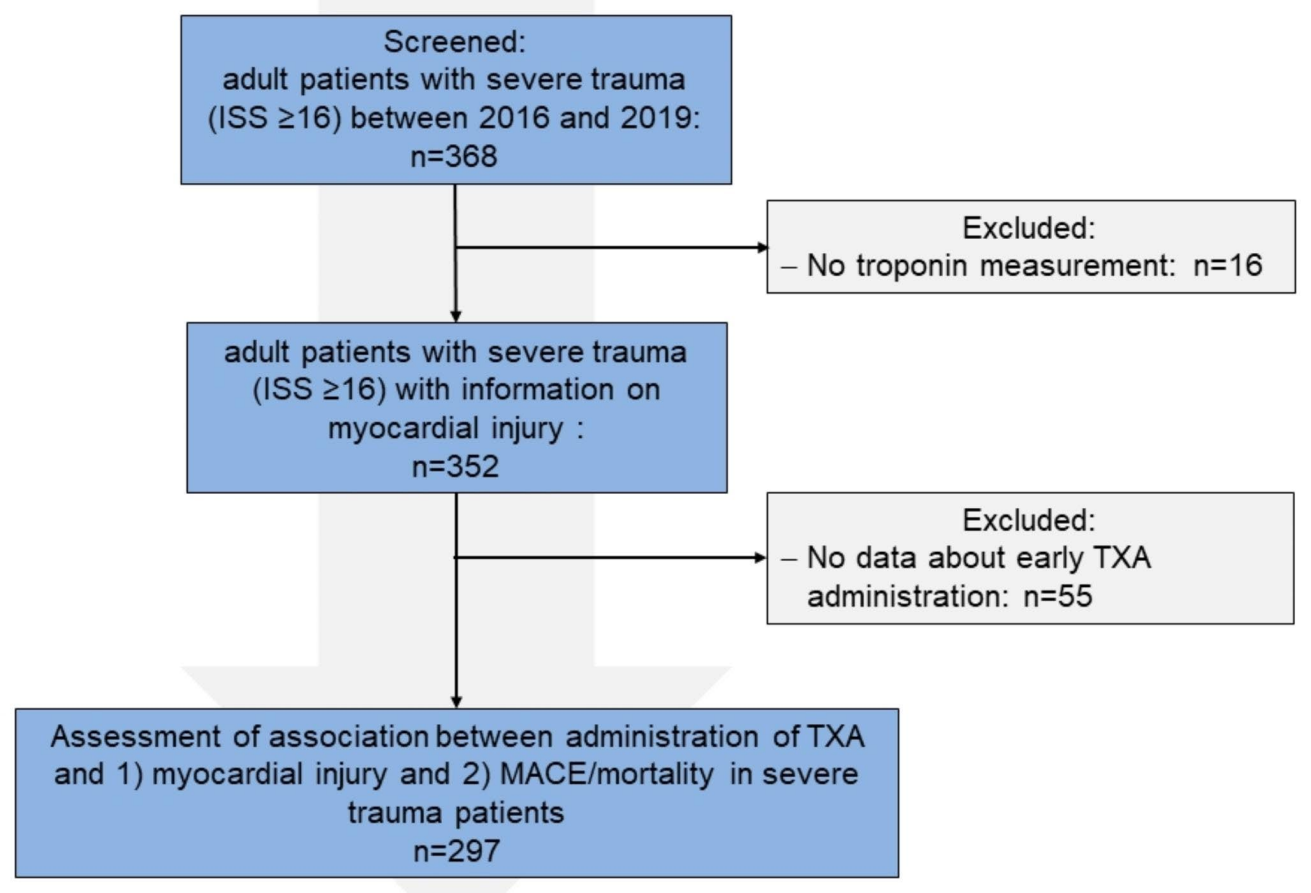
Study flow chart

We screened 368 patients for eligibility and included 297 (84%) in the final analysis (72% male, age 55 ± 21 years). The study selection process is presented in Fig. 1. 20 (7%) patients received TXA in the prehospital setting. Myocardial injury at presentation was exhibited in 119 (40%) patients. 26 (9%) developed in-hospital MACE. Mortality was 26% (76/297). Table 1 shows detailed patients characteristics for the whole cohort and for patients with and without myocardial injury at presentation.

**Table 1:** Patient characteristics

	Total cohort (n = 297)	Patients with myocardial injury at presentation (n = 119)	Patients without myocardial injury at presentation (n = 178)
*Baseline characteristics*			
Male sex no. (%)	215 (72%)	84 (71%)	131 (74%)
Age (years)	55 ± 21	62 ± 23	50 ± 19
Adipositas (Body mass index ≥ 30 kg/m²)	3 (1%)	2 (2%)	1 (1%)
*Comorbidities*			
Coronary artery disease	21 (7%)	10 (8%)	11 (6.0%)
Chronic kidney disease (≥ CKD III)	5 (2%)	4 (3%)	1 (1%)
Diabetes mellitus	17 (6%)	6 (5%)	11 (6.0%)
History of arterial hypertension	70 (24%)	35 (29%)	35 (20%)
Peripheral artery desease	3 (1%)	1 (1%)	2 (1%)
*Trauma related data*			
ISS	27 ± 11 25 [18–33]	30 ± 11 25 [21–34]	25 ± 10 22 [17–29]
GCS at ED arrival	10 ± 5 3 [3–15]	8 ± 5 7 [3–14]	11 ± 5 13 [4.5–15]
Thorax trauma	150 (50.5%)	63 (53%)	87 (49%)
*Laboratory values*			
Hb (g/dl)	12.2 ± 2.4	11.6 ± 2.6	12.7 ± 2.2
INR	1.4 ± 0.8	1.5 ± 1.0	1.2 ± 0.6
PTT (sec)	31.8 ± 25.0	38.7 ± 34.0	27.5 ±15.7
Base excess	-3.5 ± 5.5	-5.5 ± 6.6	-2.3 ± 4.2
HsTnT initial (ng/l)	56.8 ± 444.7 10.0[6.0–26.0]	130.8 ± 697.7 69.0 [39.0-161.0]	7.2 ± 2.9 7.0 [5.0–9.0]
Creatinine initial (mg/dl)	1.0 ± 0.36 1.0 [0.8–1.13]	1.13 ±0.45 1.1 [0.9–1.3]	0.9 ± 0.24 0.9 [0.73–1.06]
*Coagulation Therapy*			
TXA prehospital	20 (7%)	6 (5.0%)	14 (8%)
TXA in-hospital	81 (27%)	35 (29%)	46 (26%)
FFP	62 (21%)	32 (27%)	30 (70%)
PPSB	32 (11%)	18 (15%)	14 (8.0%)
Factor XIII	13 (5%)	5 (4%)	8 (5.0%)
Fibrinogen	39 (13%)	21 (18%)	18 (10%)
Calcium	41 (14%)	22 (19%)	19 (11.0%)
*Outcome*			
Death in hospital	76 (26%)	55 (46%)	21 (12%)
Myocardial injury at day 1	97/127 (76%)	63/63 (100%)	34/64 (53%)
Myocardial injury at day 2	66/89 (74%)	47/47 (100%)	19/42 (45%)
In-hospital MACE	26 (9%)	17 (14%)	9 (5%)
Non-fatal cardiac arrest	15 (5%)	12 (10%)	3 (2%)
Myocardial infarction	1 (0.3%)	1 (1%)	0 (0%)
New onset arrhythmias	8 (3%)	4 (3%)	4 (2%)
stroke	5 (2%)	3 (3%)	2 (1%)
AKI^1^	33 (11%)	21 (18%)	12 (7%)

*Values are presented as N (%) or Mean (± SD) / Median (IQL), where appropriate;^1^ 109 missing values ASA = American Society of Anesthesiologists; ISS = Injury Severity Score; ED = emergency department; GCS = Glasgow Coma Scale; Hb = Haemoglobin; INR = International Normalized Ratio; PTT = Partial Thromboplastin Time; HsTnT = High Sensitive Troponin, TXA = tranexamic acid, MACE = major advesre cardiac events, AKI = acute kidney injury

### Association between prehospital administration of TXA and myocardial injury

Univariate Odds ratio (OR) for the association between prehospital TXA administration and myocardial injury at presentation was 0.62 (95%CI: 0.23–1.67). After forced entry of predefined covariables OR was 0.75 [95% confidence interval (CI): 0.25–2.23]. Full results of the multivariate logistic regression analysis for are shown in Table 2.

**Table 2:** Multivariate logistic regression model for prehospital administration of TXA and myocardial injury at presentation, inhospital MACE and inhospital mortality

Outcome measure	Variable	Regression Coefficient	Odds Ratio (95% CI)
**Myocardial injury at presentation**	**TXA administration**	**-0.291**	**0.75 (0.25–2.23)**
	Age per year	0.034	1.04 (1.02–1.05)
	Sex	-0.172	0.84 (0.46–1.55)
	Hypotension (MAP < 65mmHg)	0.201	1.22 (1.07–1.40)
	ISS	0.014	1.01 (1.0-1.04)
	Coronary artery disease	-0.118	0.90 (0.33–2.42)
	Diabetes mellitus	-0.211	0.81 (0.26–2.55)
	Thorax trauma	0.335	1.40 (0.80–2.45)
	Hemoglobin	-0.121	0.89 (0.79–0.997)
**Inhospital MACE**	**TXA administration**	**-0.672**	**0.51 (0.06–4.30)**
	Age per year	0.016	1.02 (0.996–1.04)
	Sex	-0.854	0.43 (0.14–1.32)
	Hypotension (MAP < 65mmHg)	0.277	1.32 (1.15–1.52)
**Inhospital mortality**	**TXA administration**	**-0.178**	**0.84 (0.21–3.33)**
	Age per year	0.051	1.05 (1.03–1.07)
	Sex	-0.046	0.96 (0.48–1.92
	Hypotension (MAP < 65mmHg)	0.358	1.43 (1.23–1.67)
	ISS	0.057	1.06 (1.03–1.09)
	Coronary artery disease	0.307	1.36 (0.46–4.02
	Diabetes mellitus	-0.120	0.89 (0.23–3.48)
	Thorax trauma	-0.848	0.43 (0.21–0.88)

TXA = tranexamic acid, ISS = injurity severity score, MAP = mean arterial pressure

### Sensitivity analysis

In order to test the validity of the above-mentioned multivariate logistic regression model, we performed several sensitivity analyses. Changing the independent variable to early administration of TXA, defined as administration either in the prehospital setting or in the resuscitation room, the adjusted OR for the association between administration of TXA and myocardial injury was 1.04 [95%CI: 0.58–1.89].

Full results of sensitivity analysis are shown in supplementary Tables 1–5.

### Association between prehospital administration of TXA and MACE/mortality

Multivariate logistic regression analysis with forced entry of predefined covariables showed an OR for the association between prehospital administration of TXA and the incidence of MACE of 0.51 [95%CI: 0.06–4.30]. The adjusted OR for the association of prehospital administration of TXA with in-hospital mortality was 0.84 [0.21–3.33]. Full results of multivariate logistic regression analysis can be found in Table 2.

Adjusted OR for the association of early administration of TXA and MACE / mortality can be found in supplemental Tables 4 and 5.

## Discussion

The main findings of this analysis are firstly, that prehospital administration of TXA in severely injured patients did not affect the incidence of myocardial injury in this cohort. Secondly, early administration of TXA is presumably not associated with the incidence of MACE in patients with severe trauma.

### Comparison to previous studies

To date, evidence about the association of TXA administration and the incidence of myocardial injury is limited as most studies investigated the association of TXA with mortality and vasoocclusive events such as myocardial infarction.

The CRASH-2 trial investigated the effect of early administration of TXA on death, vascular occlusive events and the receipt of blood transfusion in 20,211 trauma patients [2]. In this study early administration of TXA reduced in-hospital all-cause mortality and was not associated with the incidence of myocardial infarction. Also other vascular occlusive events were not associated with the administration of TXA in this cohort. Nevertheless, the study population differed slightly from ours, as adult trauma patients with significant hemorrhage [systolic blood pressure (BP) < 90 mmHg, heart rate > 110 beats per minute or both] were included in the study. There are no data on injury severity scores. Data collection, i.e. the occurrence of myocardial infarction was recorded by an outcome form. No troponin values are reported.

Neeki et al. examined 724 patients in the Cal-PAT study and evaluated whether the administration of TXA is associated with mortality in patients with severe trauma [12]. Their findings suggest that TXA is associated with lower mortality in this cohort. There was no difference in adverse events including myocardial infarction, but they did not investigate the incidence of myocardial injury.

The findings of the STAAMP trial by Guyette et al., investigating 927 trauma patients, are as follows: administration of TXA was not associated with reduced mortality in severe trauma patients at risk for hemorrhage, which is in line with our results [13]. Further, they could not find an increased rate of vasoocclusive events, i.e. myocardial infarction, but also did not have data on troponin values and myocardial injury.

In 1310 trauma patients, prehospital TXA administration was associated with reduced mortality after 28 days [Risk ratio (RR): 0.79 (95%CI:0.63–0.99)], but not after 6 months [RR: 0.83 (95%CI: 0.67–1.03]. Moreover, it was not associated with vascular occlusive events, especially myocardial infarction [RR: 1.95 (95%CI: 0.59–6.45]. No information is given about myocardial injury [14].

In the non-cardiac surgery setting, Devereaux et al. investigated in 9,535 patients whether tranexamic acid is associated with bleeding or cardiac complications, including myocardial infarction or isolated ischemic troponin elevation. They could not show noninferiority of tranexamic acid to placebo regarding the composite cardiovascular outcome and Hazard ratio for the incidence of 30-day myocardial injury was 1.02 (95%CI: 0.91–1.14) [5].

Against this background, the results of our study can be discussed as follows: our main outcome is the incidence of myocardial injury regardless of its cause (i.e. not necessarily occlusive genesis, but also due to oxygen demand/supply imbalance), which is underrepresented in the previous studies. Nevertheless, our results match with the findings above, as TXA administration was not associated with myocardial injury in our cohort. Accumulating evidence suggests an association between myocardial injury, MACE, and mortality, in particular in non-cardiac surgery patients, but also in patients with severe trauma [15, 16]. Thus, our findings underline with special regard to myocardial injury, TXA administration seems not to be detrimental in this cohort. As our study population is defined as severely injured patients (ISS ≥16) regardless of the risk for hemorrhage, one could conclude, that the administration of TXA seems to be safe, although in patients probably not at high risk for bleeding complications.

### Limitations

We are aware that our study has several limitations: Firstly, TXA administration (i.e. dosing, time of administration, indication) and follow-up troponin measurements on days one and two were not according to a protocol due to the retrospective study design. This might have led to selection bias. I.e. patients with elevated troponin at presentation were more likely to receive follow-up measurements and thus patients developing myocardial injury on day 1 or 2 might be underrepresented. In the examined population, the application of tranexamic acid was carried out according to clinical indication based on the recommendations of the national guideline for the treatment of patients with polytrauma [1]. Unfortunately, there are no precise data about when exactly and in what dose the application took place. However, the hospital infrastructure around our center offers short distances and the local standard operating procedure (SOP) recommends 1 g of TXA in the preclinical setting. Therefore, one can assume that in most cases of documented administration, 1 g was administered within 3 h after trauma. We have no valid information about the incidence of severe head injuries or the incidence of hyperfibrinolysis as potential confounders after the application of TXA. Moreover, we had no information on prehospital risk factors for hemorrhage (e.g. blood pressure or heart rate) and thus could not certainly identify patients at high risk for bleeding. This could explain why only a relatively small proportion of 32% (and only 7% in prehospital setting) received TXA. These were possibly the patients in whom life-threatening bleeding was assumed. Bias was reduced by multivariable adjustment of the regression analysis and sensitivity analyses. Secondly, we could only refer to a comparatively small study group of 297 patients.

## Conclusions

In patients with severe trauma, early administration of TXA was not associated with myocardial injury. TXA did also not affect incidence of in-hospital MACE. These findings may underline the cardiovascular safety of early administration of TXA in the trauma setting.

### Electronic supplementary material

Below is the link to the electronic supplementary material.

Supplementary Material 1

## Data Availability

The datasets generated during and/or analysed during the current study are available from the first author on reasonable request.
