# Supplementary material for: Possible effect of the early administration of tranexamic acid on myocardial injury in patients with severe trauma
Source: J Thromb Thrombolysis. 2023 Oct 15;57(2):179–85. doi: 10.1007/s11239-023-02898-4 (PMC10869375; doi:10.1007/s11239-023-02898-4)
Supplement: Supplementary file 1 — Supplementary Material 1 [file 11239_2023_2898_MOESM1_ESM.docx]

**Supplementary Material: Effect of the administration of tranexamic acid on myocardial injury in patients with severe trauma**

Journal of Thrombosis and Thrombolysis

*Alexandra Stroda, Simon Thelen, René M’Pembele, Theresa Tenge, Carina Jaekel, Erik Schiffner, Dan Bieler, Michael Bernhard, Ragnar Huhn, Giovanna Lurati Buse, Sebastian Roth*

**Address for correspondence:**

Carina Jaekel, MD

Department of Orthopedics and Trauma Surgery

University Hospital Duesseldorf

Moorenstr. 5, 40225 Duesseldorf, Germany

Fon: +49 (0)211 81-04400

Email: [Carina.Jaekel@med.uni-duesseldorf.de](mailto:Carina.Jaekel@med.uni-duesseldorf.de)

**Supp. Table 1: Multivariate logistic regression model for early administration of TXA and myocardial injury at presentation**

| **Variable** | **Regression Coefficient** | **Odds Ratio (95% CI)** | **p-value** |
| --- | --- | --- | --- |
|  |  |  |  |
| TXA administration | 0.041 | 1.042 (0.58-1.89) | 0.89 |
| Age per year | 0.034 | 1.035 (1.02-1-05) | <0.001 |
| Sex | -0.163 | 0.85 (0.46-1.56) | 0.6 |
| Hypotension (MAP<65mmHg) | 0.2 | 1.22 (1.07-1.4) | 0.004 |
| ISS | 0.014 | 1.02 (0.99-1.04) | 0.299 |
| Coronary artery disease | -0.134 | 0.88 (0.32-2.37) | 0.79 |
| Diabetes mellitus | -0.218 | 0.80 (0.26-2.52) | 0.70 |
| Thorax trauma | 0.341 | 1.41 (0.80-2.46) | 0.233 |
| Hemoglobin | -0.119 | 0.89 (0.79-1.00) | 0.05 |

TXA=tranexamic acid, ISS=Injurity severity score

**Supp. Table 2: Multivariate logistic regression model for early administration of TXA and myocardial injury at day 1**

| **Variable** | **Regression Coefficient** | **Odds Ratio (95% CI)** | **p-value** |
| --- | --- | --- | --- |
|  |  |  |  |
| TXA administration | 0.750 | 2.12 (0.66-6.82) | 0.21 |
| Age per year | 0.035 | 1.04 (1.01-1.07) | 0.01 |
| Sex | -1.385 | 0.25 (0.08-0.82) | 0.02 |
| Hypotension (MAP<65mmHg) | 0.180 | 1.20 (0.91-1.57) | 0.20 |
| ISS | 0.029 | 1.03 (0.96-1.10) | 0.40 |
| Coronary artery disease | 0.026 | 1.03 (0.10-10.48) | 0.98 |
| Diabetes mellitus | 1.021 | 2.78 (0.29-26.33) | 0.37 |
| Thorax trauma | 0.840 | 2.32 (0.85-6.30) | 0.10 |
| Hemoglobin | -0.192 | 0.83 (0.65-1.05) | 0.12 |

TXA=tranexamic acid, ISS=Injurity severity score

**Supp. Table 3: Multivariate logistic regression model for early administration of TXA and myocardial injury at day 2**

| **Variable** | **Regression Coefficient** | **Odds Ratio (95% CI)** | **p-value** |
| --- | --- | --- | --- |
|  |  |  |  |
| TXA administration | 0.507 | 1.66 (0.50-5.54) | 0.40 |
| Age per year | 0.052 | 1.05 (1.02-1.09) | 0.001 |
| Sex | -0.669 | 0.51 (0.14-1.83) | 0.30 |
| Hypotension (MAP<65mmHg) | 0.149 | 1.16 (0.86-1.57) | 0.34 |
| ISS | 0.015 | 1.02 (0.95-1.08) | 0.65 |
| Coronary artery disease | 19.003 | 178944824.56 (0.0-x) | 0.999 |
| Diabetes mellitus | -0.213 | 0.81 (0.12-5.31) | 0.83 |
| Thorax trauma | 0.850 | 2.34 (0.71-7.70) | 0.16 |
| Hemoglobin | -0.120 | 0.89 (0.68-1.18) | 0.41 |

TXA=tranexamic acid, ISS=Injurity severity score

**Supp. Table 4: Multivariate logistic regression model for early administration of TXA and in-hospital MACE**

| **Variable** | **Regression Coefficient** | **Odds Ratio** | **p-value** |
| --- | --- | --- | --- |
|  |  |  |  |
| TXA administration | 0.06 | 1.82 (0.75-4.45) | 0.186 |
| Age per year | 0.19 | 1.02 (0.99-1.04) | 0.081 |
| Sex | -0.832 | 0.44 (0.14-1.36) | 0.151 |
| Hypotension (MAP<65mmHg) | 0.252 | 1.29 (1.11-1.49) | 0.001 |

TXA=tranexamic acid

**Supp. Table 5: Multivariate logistic regression model for early administration of TXA and mortaility**

| **Variable** | **Regression Coefficient** | **Odds Ratio** | **p-value** |
| --- | --- | --- | --- |
|  |  |  |  |
| TXA administration | -0.063 | 0.94 (0.46-1.94) | 0.865 |
| Age per year | 0.051 | 1.05 (1.03-1.07) | <0.001 |
| Sex | -0.044 | 0.96 (0.48-1.92) | 0.902 |
| Hypotension (MAP<65mmHg) | 0.361 | 1.43 (1.23-1.68) | <0.001 |
| ISS | 0.058 | 1.06 (1.03-1.09) | <0.001 |
| Coronary artery disease | 0.298 | 1.35 (0.46-3.99) | 0.59 |
| Diabetes mellitus | -0.136 | 0.87 (0.22-3.42) | 0.87 |
| Thorax trauma | -0.851 | 0.43 (0.21-0.88) | 0.02 |

TXA=tranexamic acid, ISS=Injurity severity score
